# Supplementary material for: P-TRAP: a Panicle Trait Phenotyping tool
Source: BMC Plant Biol. 2013 Aug 29;13:122. doi: 10.1186/1471-2229-13-122 (PMC3848748; doi:10.1186/1471-2229-13-122)
Supplement: Additional file 7 — P-TRAP source code. The Netbeans project that contains the source code of the application. [file 1471-2229-13-122-S7.zip › javadoc.zip!/javadoc/org-bioalg-graphprocessor/org/bioalg/graphprocessor/Edge.html]

Edge (GraphProcessor)


JavaScript is disabled on your browser.


- Package
- Class
- Tree
- Deprecated
- Index
- Help

*org.bioalg.graphprocessor 1.0*

- Prev Class
- Next Class

- Frames
- No Frames

- All Classes

- Summary:
- Nested |
- Field |
- Constr |
- Method

- Detail:
- Field |
- Constr |
- Method


org.bioalg.graphprocessor

## Class Edge

- java.lang.Object
- - org.bioalg.graphprocessor.Edge

- All Implemented Interfaces:
  :   java.lang.Comparable

  ---

    

  ```
  public class Edge
  extends java.lang.Object
  implements java.lang.Comparable
  ```

  A general edge class.

  Author:
  :   faroq

- - ### Nested Class Summary

    Nested Classes

    | Modifier and Type | Class and Description |
    | --- | --- |
    | `static class` | `Edge.EDGE_TYPE` |
  - ### Field Summary

    Fields

    | Modifier and Type | Field and Description |
    | --- | --- |
    | `double` | `info` |
  - ### Constructor Summary

    Constructors

    Constructor and Description

    `Edge(java.awt.Point p1,
    java.awt.Point p2)`
  - ### Method Summary

    Methods

    | Modifier and Type | Method and Description |
    | --- | --- |
    | `int` | `compareTo(java.lang.Object t)` |
    | `boolean` | `equals(java.lang.Object o)` |
    | `java.awt.Point` | `getP1()` |
    | `java.awt.Point` | `getP2()` |
    | `Edge.EDGE_TYPE` | `getType()` |
    | `void` | `setP1(java.awt.Point p1)` |
    | `void` | `setP2(java.awt.Point p2)` |
    | `void` | `setType(Edge.EDGE_TYPE type)` |

    - ### Methods inherited from class java.lang.Object

      `clone, finalize, getClass, hashCode, notify, notifyAll, toString, wait, wait, wait`

- - ### Field Detail


    - #### info

      ```
      public double info
      ```
  - ### Constructor Detail


    - #### Edge

      ```
      public Edge(java.awt.Point p1,
          java.awt.Point p2)
      ```
  - ### Method Detail


    - #### getP1

      ```
      public java.awt.Point getP1()
      ```


    - #### setP1

      ```
      public void setP1(java.awt.Point p1)
      ```


    - #### getP2

      ```
      public java.awt.Point getP2()
      ```


    - #### setP2

      ```
      public void setP2(java.awt.Point p2)
      ```


    - #### getType

      ```
      public Edge.EDGE_TYPE getType()
      ```


    - #### setType

      ```
      public void setType(Edge.EDGE_TYPE type)
      ```


    - #### equals

      ```
      public boolean equals(java.lang.Object o)
      ```

      **Overrides:**
      :   `equals` in class `java.lang.Object`


    - #### compareTo

      ```
      public int compareTo(java.lang.Object t)
      ```

      **Specified by:**
      :   `compareTo` in interface `java.lang.Comparable`


- Package
- Class
- Tree
- Deprecated
- Index
- Help

*org.bioalg.graphprocessor 1.0*

- Prev Class
- Next Class

- Frames
- No Frames

- All Classes

- Summary:
- Nested |
- Field |
- Constr |
- Method

- Detail:
- Field |
- Constr |
- Method
